# A Study of Spontaneous Self-Injurious Behavior and Neuroimaging in Rhesus Macaques

**DOI:** 10.34133/research.0782

**Published:** 2025-07-31

**Authors:** Ya-Li Zhang, Yi-Cheng Qiao, Yun-Chao Ji, Hong-Di Huang, Long Zhang, Jian-Xiong Ruan, Chen-Yao Li, Hui-Heng Xie, Bao-Lin Zhang, Qi Zhou, Sha-Sha Yue, Xiao-Mei Yu, Ming-Hao Qiu, Chuan-Kai Yu, Si-Chu Wu, Yu-Fang Zhou, Yan-Ling Li, Hong-Mei Zhu, Shu-Zhen Dong, Kang Huang, Yun Wang, Qiong Wang, Yi-Jiang Li, Ya Xie, Hui-Ling Chen, Long-Bao Lv, Shu Liu, Yong-Gang Yao, Chun Wang, Ning Liu, Jian-Hong Wang

**Affiliations:** ^1^ National Research Facility for Phenotypic & Genetic Analysis of Model Animals (Primate Facility) and National Resource Center for Non-Human Primates, Kunming Institute of Zoology, Chinese Academy of Sciences, Kunming, Yunnan 650107, China.; ^2^ Yunnan Engineering Center on Brain Disease Models, Kunming Institute of Zoology, Chinese Academy of Sciences, Kunming 650107, China.; ^3^ State Key Laboratory of Cognitive Science and Mental Health, Institute of Biophysics, Chinese Academy of Sciences, Beijing 100101, China.; ^4^ College of Life Sciences, University of Chinese Academy of Sciences, Beijing 101408, China.; ^5^ School of Life Sciences, Division of Life Sciences and Medicine, University of Science and Technology of China, Hefei, Anhui 230026, China.; ^6^ State Key Laboratory of Genetic Evolution and Animal Models, Key Laboratory of Animal Models and Human Disease Mechanisms of Yunnan Province, and KIZ/CUHK Joint Laboratory of Bioresources and Molecular Research in Common Diseases, Kunming Institute of Zoology, Chinese Academy of Sciences, Kunming 650204, China.; ^7^ Yunnan Key Laboratory of Biodiversity Information, Kunming Institute of Zoology, Chinese Academy of Sciences, Kunming, Yunnan 650201, China.; ^8^ The Affiliated Brain Hospital of Nanjing Medical University, Nanjing, China.; ^9^ Guangdong Bayone Biotech CO., Ltd., Guangzhou, China.

## Abstract

Nonsuicidal self-injury (NSSI) demonstrates escalating prevalence among adolescents as a maladaptive behavior characterized by deliberate self-harm, yet its neurobiological underpinnings remain elusive. Spontaneous self-injurious behavior (SIB) in nonhuman primates (NHPs) emerges as a clinically relevant animal model for investigating NSSI pathogenesis and therapeutic interventions. However, previous studies have yet to comprehensively evaluate the translational value of self-injury in NHPs through integrated behavioral and neuroimaging characterization. In this study, we identified spontaneously self-injurious macaques within our NHP colony and performed multimodal assessments encompassing ethological profiling, neuroendocrine assays, metabolomic analysis, and neuroimaging. Our results revealed that SIB macaques exhibited biological patterns and temporal onset sequences of self-harm, accompanied by a locomotor and social interaction decrease based on 3-dimensional deep-learning recognition, sensory processing deficits, and impairments in emotional and cognitive processing. Biochemical profiling demonstrated reduced plasma concentrations of cortisol, serotonin, and oxytocin, coupled with metabolomic disturbances including up-regulation of digestive-related pathways and down-regulation of dopaminergic synaptic signaling and phosphatidylinositol metabolism. Neuroimaging analyses identified structural abnormalities featuring volumetric enlargement in amygdala and midbrain regions alongside gray matter reduction in the frontal and parietal lobes. Enhanced structural and functional connectivity was observed. Network-based statistics highlighted increased functional connectivity primarily between the frontal and parietal lobes. These alterations demonstrated a partial correlation with observed behavioral deficits in the self-injury macaques. Notably, a series of administration of low-dose ketamine had no effect on SIB and the physical index. Our integrative multi-omic approach elucidates the neurobiological phenotype of spontaneous SIB in macaques, highlighting the value of the SIB model for pathogenetic investigation and therapeutic development in human NSSIs.

## Introduction

Nonsuicidal self-injury (NSSI) disorder, classified as a distinct clinical condition in *Diagnostic and Statistical Manual of Mental Disorders—Fifth Edition* (DSM-5), involves deliberate self-harm without suicidal intent, often manifesting as cutting, burning, or hitting to induce pain or tissue damage. Prevalence ranges from 1% to 4% in adults to 15% to 18% in adolescents, with underdiagnosis potentially elevating real-world rates [1,2]. Triggered by emotional distress or interpersonal conflicts, NSSI individuals may exhibit functional heterogeneity across emotional regulation, attention-seeking, and social avoidance [3]. DSM-5 criteria specify that NSSI behaviors aim to (a) alleviate negative emotions/cognitions, (b) resolve relational difficulties, or (c) generate positive feelings. In addition, NSSI shows heterogenetic traits, e.g., intellectual disability, aggression, and impulsivity, and frequently co-occurs with borderline personality disorder, major depressive disorders, eating disorders, and substance disorders or none [1,4]. Alarmingly, NSSI populations face elevated suicide risks despite the nonsuicidal intent [5].

NSSI arises from multifactorial etiopathogenesis involving stress-response dysfunction, genetic susceptibility, and environmental triggers. Blunted hypothalamic–pituitary–adrenal (HPA) axis activity and epigenetic glucocorticoid receptor dysregulation contribute to stress hypersensitivity, aggression, and social/sleep impairments [6]. While genome-wide studies identify suicide-related risk genes [7], specific NSSI-associated genetic markers remain undefined.

NSSI remains poorly understood, hampered by limited animal models. Valid models of self-injurious behavior (SIB) are critical for identifying the pathogenesis, therapeutic targets, and biomarkers of NSSI. Current rodent models primarily utilize monoamine agonists (e.g., pemoline) or anxiety-inducing agents (e.g., FG 7142) to induce SIB yet lack spontaneous occurrence (<5% prevalence) and human-relevant complexity in social–emotional expression [8]. These pharmacological interventions also limit translational relevance to human NSSI. Nonhuman primates (NHPs), particularly Old World macaques, offer unique advantages with 11%–15% to 1%–5% spontaneous SIB rates in captivity as the condition improved [6]. Their self-injury patterns, including limb biting, head-banging, and hairpulling, mimic human NSSI in both behavioral topography (skin wounds and tissue damage) and severity gradation. Crucially, macaques share >93% genetic homology with humans and possess homologous prefrontal cortical structures essential for emotion regulation and social cognition. This neuroanatomical fidelity enables the investigation of stress-axis dysregulation (e.g., HPA hyperactivity) and epigenetic mechanisms implicated in SIB. While genome-wide studies in rodents identify candidate pathways, NHP models uniquely capture the interplay between intrinsic vulnerabilities (genetic/epigenetic) and environmental stressors that may drive spontaneous SIB. Their complex social hierarchies and emotional repertoires permit ecologically valid studies of trigger contexts (e.g., social conflict and anxiety) absent in rodent paradigms. By bridging molecular mechanisms with clinically relevant phenotypes, NHP models may hold unparalleled potential for advancing NSSI research and therapeutic development.

This study established a novel translational framework for NSSI research by identifying spontaneously self-injurious macaques within our NHP colony. A comprehensive behavioral battery assessed locomotor patterns, social interaction, exploratory behavior, emotional and defensive responses, spatial working memory (SWM), and cognitive flexibility to map phenotypic parallels with human NSSI. We further characterized the biological rhythm and behavioral sequencing of SIB. Multimodal biomarkers were investigated through cerebrospinal fluid (CSF) metabolomics, plasma hormonal levels, structural magnetic resonance imaging (MRI), and resting-state functional MRI (rs-fMRI). Therapeutic potential was evaluated via low-dose ketamine intervention. This work provides a valid NHP model of spontaneous SIB mirroring core NSSI features and a multi-omic platform for mechanistic discovery and therapeutic development.

## Results

### Characterization of self-injury macaques

Self-injured wounds were found in self-injury macaques’ limbs reflected by mean wound scores between 3 and 58 when tested at different times. The video recordings of 10 self-injury macaques revealed that 5 of the macaques exhibited hysterical clusters of hand-biting, while the other 5 demonstrated leg-biting. The SIB occurred in the form of clusters, with the macaques repetitively engaging in self-biting. On average, a single episode of SIB lasted for 1.50 ± 0.22 s.

We found that the macaques’ SIB demonstrated sequential traits (Table). It manifested as several episodes where the macaques were mostly quiet, followed by repetitive hysterical self-biting that appeared as clusters of SIB onsets. Eventually, it ended with the macaques being mostly quiet (Movie S1) or engaging in stereotypical behavior (Movie S2) after one or several clusters of SIB. This suggested that the macaques’ SIB might be driven by impulsivity, and it seemed that the macaques experienced relief from repetitive self-injury, lending support to the hypothesis that SIB is a form of sensation seeking in response to impoverished environments [6]. It was observed that 10% aggression occurred prior to the onset of SIB, indicating that a few macaques might redirect threatening behavior toward themselves, as mentioned by Novak and Meyer [6].

**Table.** The sequential behavior of self-injury

Behavior	SIB	Prior to SIB	Post-SIB
Sitting or standing while observing outside	Single	32.61%	43.75%
	Cluster	30.00%	40.00%
Observing the self-bitten site	Single	26.09%	6.25%
	Cluster	20.00%	0.00%
Stereotypical circling	Single	21.74%	29.17%
	Cluster	20.00%	40.00%
Aggressively shaking the cage or opening the mouth	Single	15.22%	6.25%
	Cluster	10.00%	10.00%
Other behaviors (grooming or scratching head)	Single	4.35%	14.58%
	Cluster	20.00%	10.00%

SIB, self-injurious behavior

**Fig. 1.**
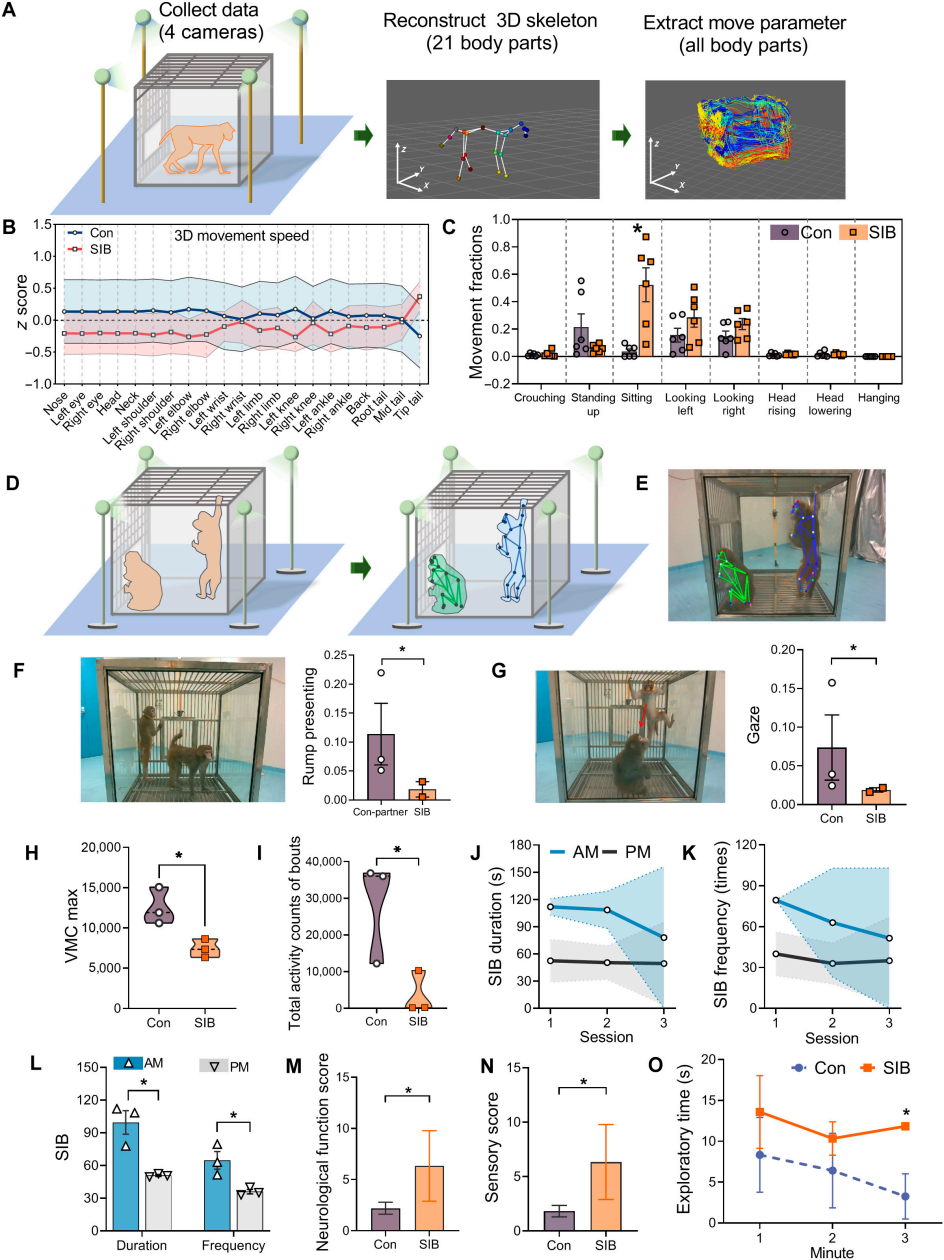
The behavioral experiments in macaques. (A) Schematic diagram of the testing dynamics of spontaneous behavior in macaques. (B and C) Comparison of movement speeds (B) and behavioral postures (C) between SIB and control macaques using 3-dimensional (3D) deep-learning-based recognition. (D) Schematic diagram of paired social interaction test. (E) Photo showing the identified macaques. (F) Macaque rump presenting and fractions between the control partner and SIB groups. (G) Macaque gaze and fractions between the control and SIB groups. The red line illustrates the macaque’s gazing direction. (H and I) Quantified using ActiGraph. Maximum vector magnitude counts (VMCs) (H) and total activity counts of bouts (I) per hour. (J to L) Duration and frequency of SIB measured in the morning (AM) and afternoon (PM). (M and N) Scores of total neurological function (M) and sensory function (N). (O) Exploratory behavior in the macaques. Data are expressed as mean ± standard error of the mean (SEM). *P < 0.05; self-injury macaques (SIB) vs. the controls (Con) or AM vs. PM.

The macaques with SIB exhibited a tendency to have a lower 3-dimensional (3D) movement speed of main body parts (head, neck, shoulder, limbs, knees, etc.) within 35 min (Fig. 1A and B). Analysis of the behavior patterns showed a significantly more sitting behavior in non-apparent displacement than the controls (*P* = 0.034) (Fig. 1C). Self-injury macaques showed overall low axial diffusivity (AD) and high non-apparent displacement when compared with the controls (Fig. S1A). Principal component analysis of kinematic parameters showed that the self-injury macaques exhibited relatively concentrated values along the first principal component (PC1) and second principal component (PC2), whereas the control animals displayed a broader range of individual variability (Fig. S1B). In the paired social interaction test (Fig. 1D and E), 2 self-injury macaques showed lower social interaction, reflected by significantly lower fractions of rump presenting (*P* = 0.023, Fig. 1F) and gaze (*P* = 0.037, Fig. 1G) to the other when compared with control partners and controls, respectively. No significant differences were found in approaching behavior. The daily activity from the ActiGraph showed that the self-injury macaques exhibited a lower level of daily locomotor activity, reflected by a significantly lower maximum vector magnitude counts than the controls (*P* = 0.03) and lower activity counts of bouts per hour (*P* = 0.04) (Fig. 1H and I). These results were consistent with previous reports where low levels of physical activity were observed in individuals with NSSIs [9].

The macaques exhibited a biological rhythm with more seizures occurring in the morning; the duration and frequency (Fig. 1J and K) of SIB tended to decrease as testing sessions increased, suggesting a slight habituation. Moreover, SIB showed a longer duration (*P* = 0.011) and a higher frequency of SIB (*P* = 0.027) in the morning than in the afternoon (Fig. 1L). It was similar to clinical studies indicating that diurnal rhythms modulated various pathophysiology and neurological disorders such as epilepsy [10].

The self-injury macaques had a serious disruptive neurological function compared with a large number of controls (*P* = 0.042, *n* = 18, Fig. 1M), and this difference was reflected by the impaired sensory function (*P* = 0.021, Fig. 1N), which mainly included the pain sensitivity test. The decreased pain sensitivity may contribute to the induction of repetitive SIB both in animals and in human NSSIs [11]. The self-injury macaques showed a longer duration of exploration to the moving laser icon than the controls (on the third minute, *P* = 0.038, Fig. 1O), suggesting that the SIB macaques had an abnormal nature exploratory behavior, probably due to sensory dysfunction.

### Macaques with SIB showed abnormal emotional responses

**Fig. 2.**
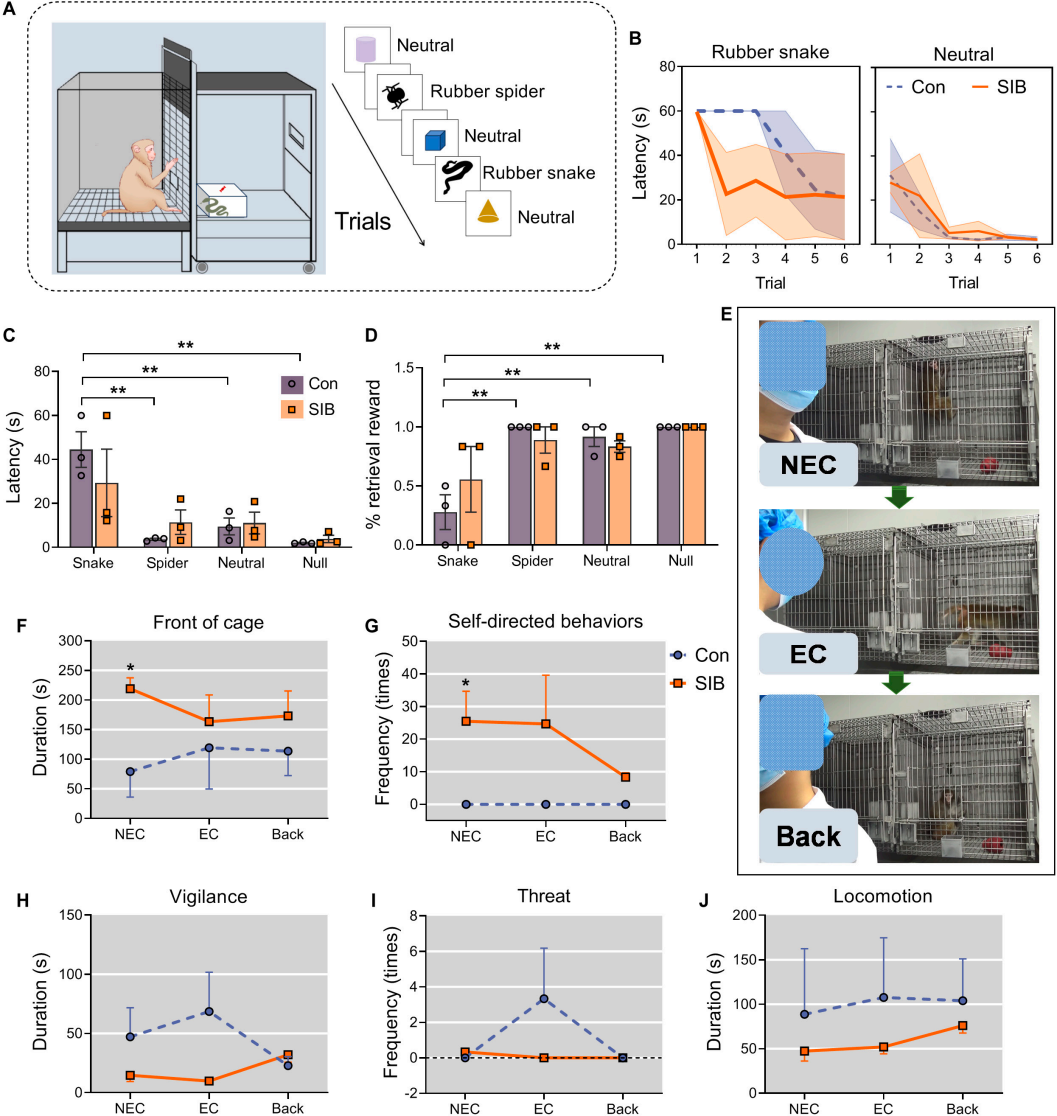
The emotional/defensive response tests in macaques. (A) Schematic diagram of emotional response test. (B to D) Behavioral results of emotional response test. The macaques with SIB tended to have a faster habituation to the rubber snake when compared with the control, and both groups showed a similar habituation to neutral stimuli (B). The latency (C) and percentage (D) for the macaques taking food rewards under different stimuli (**P < 0.01, rubber snake vs. rubber spider, neutral or null). (E) Schematic diagram of the defensive response test in the human intruder test (HIT) under 3 conditions: no eye contact (NEC), eye contact (EC), and back. (F to J) Behavioral results of HIT under different conditions. Self-injury macaques showed lower defensive behaviors, reflected by a longer-duration stay in front of the cage (F), higher self-directed (self-biting) behavior (G), a trend of lower vigilance (H), lower threat (I), and lower locomotor activity (J) when compared with the controls. Data are expressed as mean ± SEM. *P < 0.05 and **P < 0.01, self-injury macaques (SIB, *n* = 3) vs. the controls (Con, *n* = 3).

We tested the emotional responses of macaques when being challenged with different stimuli (Fig. 2A). The self-injury macaques showed quicker habituation to the rubber snake and similar habituation to neutral stimuli as the controls (Fig. 2B). Moreover, the self-injury macaques failed to show significant difference in latency (Fig. 2C) or reward retrieval (Fig. 2D) among stimuli, suggesting deficits of defensive behavior and emotional processing when compared with the control animals showing significant difference between the rubber snake and other stimuli (longer latency facing the rubber snake than facing the rubber spider [*P* < 0.001], neutral [*P* = 0.001], and null [*P* < 0.001]; took more food when the spider [*P* < 0.001], neutral [*P* = 0.001], and null [*P* < 0.001] rather the rubber snake was presented).

Similarly, in the human intruder test (Fig. 2E), macaques with SIB stayed in front of the cage for a significantly longer time than the controls at no eye contact (NEC) conditions (*P* = 0.041) (Fig. 2F), showed a higher frequency of self-directed (biting) behavior than control macaques at NEC (*P* = 0.05) conditions (Fig. 2G). However, self-injury macaques showed a tendency to have lower vigilance (Fig. 2H), threatening (Fig. 2I), and locomotor activity (Fig. 2J) depending on the position of the intruder, when compared with the control group. These results demonstrated less defensive and less anxious responses to the human intruder in the self-injury macaques.

### Macaques with SIB presented impaired SWM

**Fig. 3.**
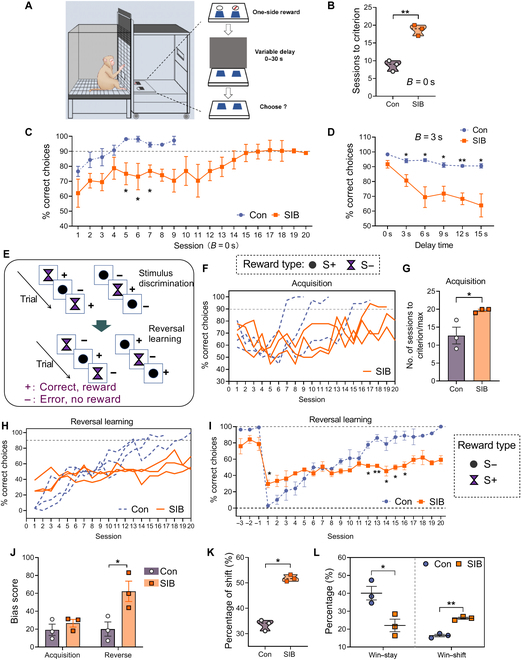
The cognitive experiments in macaques. (A) Schematic diagram of the spatial working memory (SWM) task [23]. (B and C) Results of the SWM test at *B* = 0 s. Macaques with SIB needed more sessions to obtain the criterion (B) and performed a lower percentage of correct choices than controls depending on the session (C). (D) SIB macaques showed lower performance across the delay time than controls at *B* = 3 s. (E) Schematic diagram of the reversal learning (RL) task. (F and G) Percentage of correct choices across sessions (F) and the numbers of sessions to obtain the criterion (G) during the acquisition stage. (H and I) Percentage of correct choices in the RL task. The dotted line in (C), (F), and (H) shows the criterion. (J) The bias scores in SIB and control macaques during the acquisition and RL. (K) Percentage of shifting during the RL stage. (L) Self-injury macaques showed a significantly lower percentage of win–stay and a higher percentage of win–shift than the controls. Data are expressed as mean ± SEM. *P < 0.05 and **P < 0.01, self-injury macaques. SIB, *n* = 3; Con, *n* = 3.

When the macaques were trained in the SWM task (Fig. 3A), macaques with SIB needed more sessions to obtain the criterion at *B* = 0 s than controls (*P* = 0.001) (Fig. 3B). The fast learner control macaque spent 7 sessions to obtain the criterion when *B* = 0 s. Thus, we analyzed the performance of 7 sessions between 2 groups and found a significantly lower percentage of correct choices in self-injury macaques than controls at session 5 (*P* = 0.035), session 6 (*P* = 0.05), and session 7 (*P* = 0.015) (Fig. 3C). When *B* = 3 s (Fig. 3D), the performance decreased as the delay time increased (overall delay time effect *F*(5,20) = 15.09, *P* < 0.001), and this decrease was significantly faster in self-injury macaques (interaction of group × delay time *F*(5,20) = 5.21, *P* = 0.003; between-group effect *F*(1,4) = 17.01, *P* = 0.015). The correct choices were significantly lower in SIB than in controls at delay times of 3 s (*P* = 0.034), 6 s (*P* = 0.024), 9 s (*P* = 0.021), 12 s (*P* = 0.005), and 15 s (*P* = 0.028).

In the reversal learning (RL) task (Fig. 3E), 2 self-injury macaques failed to obtain the criterion at the acquisition of discrimination training, resulting in a significantly higher number of sessions in the SIB group than in the control group (*P* = 0.041, Fig. 3F and G). After the reward was reversed from the solid cycle into the diamond, the macaques gradually learned the new rule (overall session effect *F*(19,38) = 23.51, *P* < 0.001), and this learning was significantly slower in self-injury macaques (interaction of group × session *F*(19,38) = 7.10, *P* < 0.001). The self-injury macaques failed to obtain the criterion (Fig. 3H). As shown in Fig. 3I, significantly lower performances were found in the SIB group than in controls at session 12 (*P* = 0.004), session 13 (*P* = 0.001), session 14 (*P* = 0.03), session 15 (*P* = 0.011), and session 16 (*P* = 0.016). These findings were consistent with previous studies indicating that cognitive risk factors have associations with the functions and severity of SIB in the macaque model and NSSI [12,13]. However, the SIB group showed a higher performance than controls at session 1 (*P* = 0.004) when the reward was reversed, suggesting an abnormal behavior.

Similarly, we measured the bias score, which may reflect a stereotypical behavior, and the switch score in the win–stay–lose–shift paradigm [14], which may represent impulsivity, and found self-injury macaques significantly increased the bias to choose one side after the reward stimulus shifted to the opposite of the previous rule (*P* = 0.039, as shown in Fig. 3J) although there was no significant difference in bias score between SIB and control macaques during the acquisition. Moreover, the self-injury macaques showed a significantly higher percentage of win–shift and lose–shift than the controls during the RL process (*P* = 0.0002) (Fig. 3K). In detail, the self-injury macaques presented a significantly lower percentage of win–stay (*P* = 0.025) and a higher percentage of win–shift (*P* = 0.0004), and also a tendency toward a shorter reaction time, when compared with the controls (Fig. 3L). These findings were consistent with the pathogenesis hypothesis that compulsivity and impulsivity might be an implicit cause of SIB, which has also been observed in NSSI [15].

### Self-injury macaques showed lower levels of cortisol, serotonin, and oxytocin in plasma and dysregulated metabolomic patterns in CSF

**Fig. 4.**
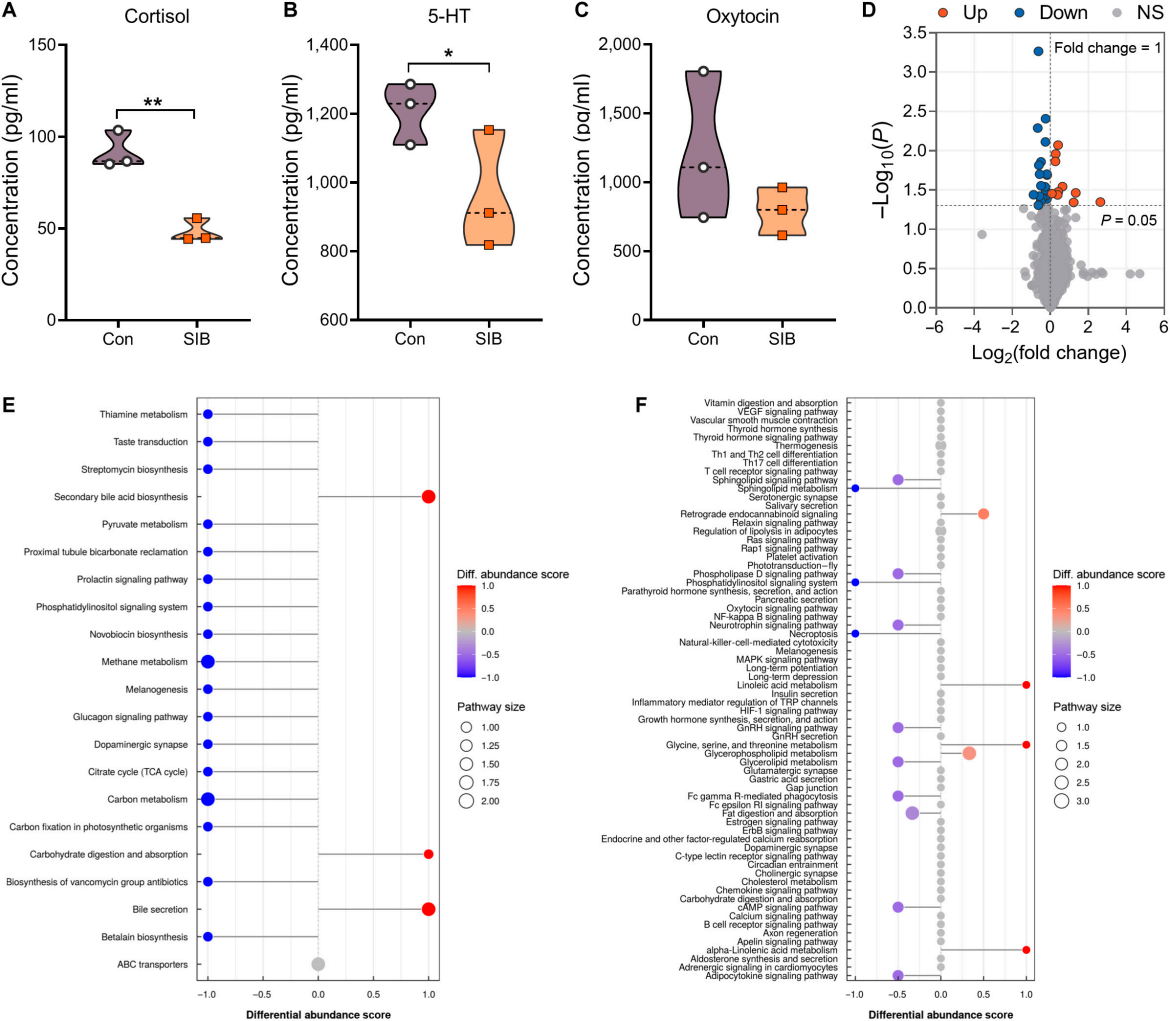
The plasma hormone level and cerebrospinal fluid (CSF) metabolomic pattern in macaques. (A to C) Plasma concentrations of cortisol, serotonin (5-HT), and oxytocin in SIB macaques versus controls. (D) Volcano plot illustrating the metabolites with significant differences. Up (red dots), up-regulation; Down (blue dots), down-regulation; NS (gray dots), no significant difference. (E and F) Plots showing the differential abundance score of differential Kyoto Encyclopedia of Genes and Genomes (KEGG) pathways in hydrophilic metabolites (E) and lipids (F) in CSF. Data are expressed as mean ± SEM. *P < 0.05; **P < 0.01. SIB, *n* = 3; Con, *n* = 3.

Considering the impact of hormones on SIB, the emotion-related hormones in peripheral circulation were measured. The self-injury macaques had significantly lower cortisol (*P* = 0.003) and serotonin (5-HT) (*P* = 0.046) and a trend toward lower oxytocin in the plasma when compared with the control animals (Fig. 4A to C), which were partly consistent with the findings of clinical and animal studies [6,16,17].

Furthermore, metabolomic analysis showed that 969 metabolites had been determined in the macaques. We identified a total of 30 differential metabolites between the SIB and control groups, including 20 down-regulated metabolites, such as myo-inositol and arachidic acid, and 10 up-regulated metabolites, including phosphatidylcholine, triglycerides, and diacylglycerol (Fig. 4D). In detail, 9 differential metabolites were hydrophilic metabolites and the other 21 differential metabolites were lipids (Tables S1 and S2). We next performed Kyoto Encyclopedia of Genes and Genomes pathway enrichment analysis and calculated the differential abundance scores of differential pathways separately on CSF hydrophilic metabolites and lipids. Results showed that pathways related to carbohydrate digestion were notably up-regulated; however, dopaminergic synapse activity and the phosphatidylinositol signaling pathway were significantly down-regulated in self-injury macaques when compared with those of the controls.

### Self-injury macaques had significant brain structural changes compared with control macaques

**Fig. 5.**
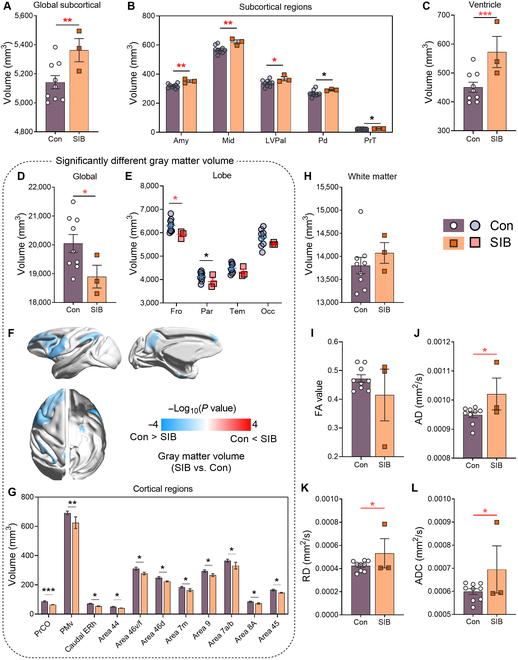
Differences in brain structure between SIB macaques and controls. (A to C) Volume of subcortical structures (A), detailing subcortical regions (B) and ventricle (C) with significant differences between SIB and control macaques. Amy, amygdala; Mid, midbrain; LVPal, lateral and ventral pallia; Pd, pallidum; PrT, pretectum. (D and E) Gray matter volume (GMV) at the whole-brain level (D) and the lobe level (E). Fro, frontal lobe; Par, parietal lobe; Tem, temporal lobe; Occ, occipital lobe. (F) Visualization of the cortical regions in (G) on the mid-gray surfaces of the macaque template. Red indicates regions where self-injury macaques had a larger GMV than controls, while blue indicates the opposite. (G) The GMV in cortical regions with significant differences between macaques with SIB and controls. PrCO, precentral operculum; PMv, ventral premotor cortex. (H to L) Bar plots representing the volume (H), fractional anisotropy (FA) (I), axial diffusivity (AD) (J), radial diffusivity (RD) (K), and apparent diffusion coefficient (ADC) (L) of white matter (WM) at the whole-brain level in self-injury macaques versus controls. *P < 0.05; **P < 0.01; ***P < 0.001; red asterisks indicate false discovery rate (FDR)-corrected *P* values: *P < 0.05; **P < 0.01; ***P < 0.001. SIB, *n* = 3; Con, *n* = 9.

We used a general linear mixed model (GLMM) to compare the subcortical volumes of macaques exhibiting SIB with those of the controls. Our analysis revealed that the self-injury macaques had significantly larger subcortical volumes than the controls (*P* = 0.002) (Fig. 5A). To pinpoint which specific subcortical structures contributed to this overall difference, we performed GLMM analyses on 13 defined subcortical structures. We found that 5 of these structures were significantly larger in the self-injury macaques than in the controls (uncorrected *P* < 0.05), including the amygdala (*P* = 0.002), the midbrain (*P* = 0.002), lateral and ventral pallium (*P* = 0.038), pallidum (uncorrected *P* = 0.024), and pretectum (uncorrected *P* = 0.029) (Fig. 5B). Additionally, the ventricles were significantly enlarged in self-injury macaques compared with those in the control animals (*P* < 0.001) (Fig. 5C).

We employed a GLMM to compare gray matter volumes (GMVs) between SIB and control macaques. Our analysis revealed a significant reduction in whole-brain GMV in the self-injury macaques (*P* = 0.010) (Fig. 5D). To gain a deeper understanding of GMV changes in self-injury macaques, we divided the cortical surfaces into 4 lobes and 88 regions using the CHARM1 and CHARM5 templates. Our analysis revealed significant reductions in GMV in both the frontal lobe (*P* = 0.017) and the parietal lobe (uncorrected *P* = 0.035) (Fig. 5E). Within the frontal lobe, notable decreases were observed in the ventrolateral prefrontal cortex (PFC) across area 44 (uncorrected *P* = 0.011), area 46v/f (uncorrected *P* = 0.022), and area 45 (uncorrected *P* = 0.050); the dorsolateral PFC in area 46d (uncorrected *P* = 0.028) and area 9 (uncorrected *P* = 0.041); the orbitofrontal cortex (OFC) at the precentral operculum (uncorrected *P* < 0.001); the frontal eye field area 8A (uncorrected *P* = 0.050); and the motor cortex at the ventral premotor cortex (uncorrected *P* = 0.008). In addition, we identified significant volume reductions in the temporal lobe at the caudal entorhinal cortex (ERh) (uncorrected *P* = 0.011) and in the parietal lobe at areas 7m (uncorrected *P* = 0.031) and 7a/b (uncorrected *P* = 0.041) (Fig. 5F and G).

We also quantified potential changes in white matter (WM) in macaques with SIB, but we did not find significant differences in WM volume between the SIB and the control group (*P* = 0.243, Fig. 5H). However, we indeed observed significantly higher values in the self-injury macaques for the diffusion tensor imaging scalar metrics: AD (*P* = 0.014), radial diffusivity (*P* = 0.035), and apparent diffusion coefficient (*P* = 0.026) (Fig. 5I to L). These results suggest potential microstructural changes in the WM of the self-injury macaques.

### Macaques with SIB showed enhancement of structural connectivity and functional connectivity

**Fig. 6.**
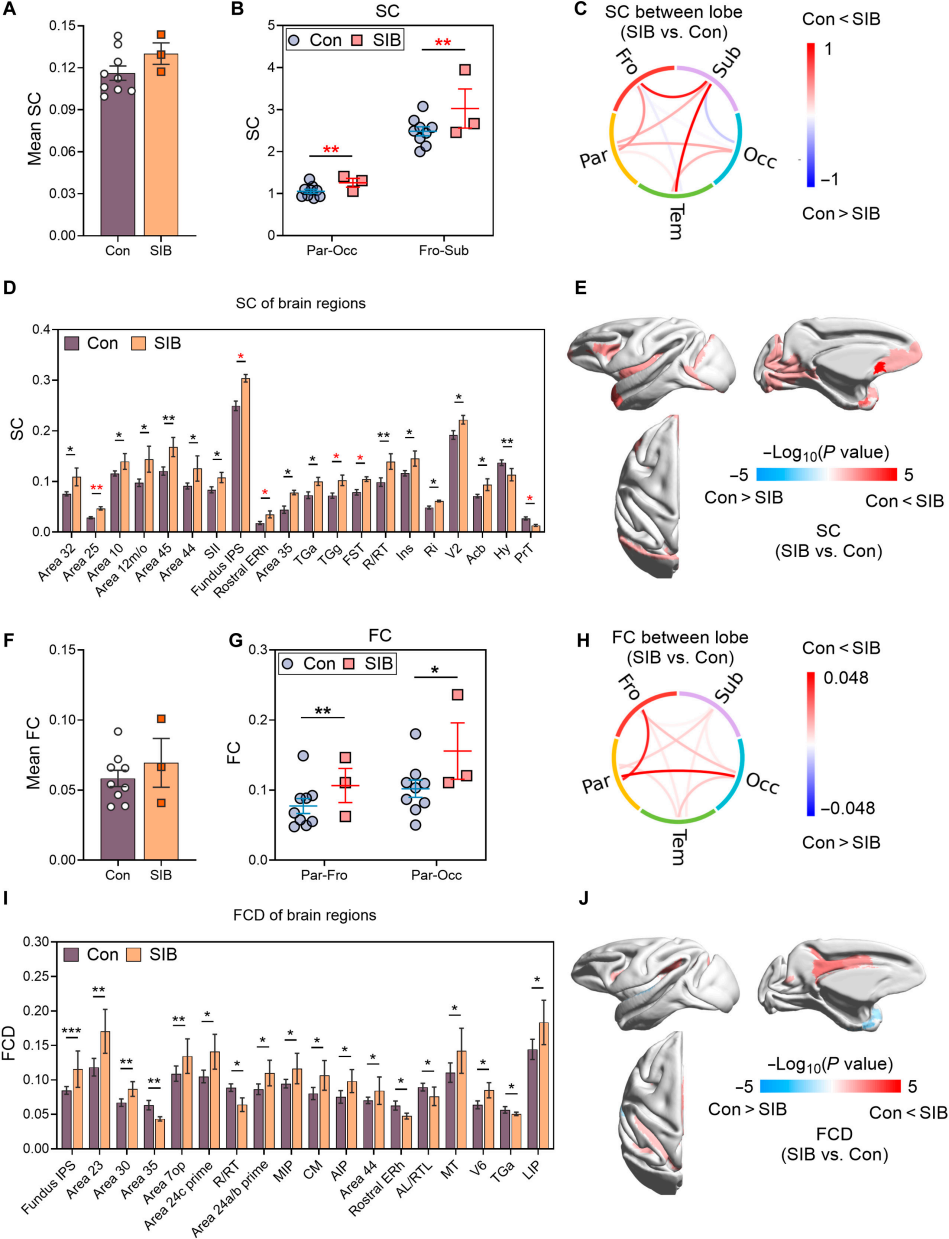
Differences in structural connectivity (SC) and functional connectivity (FC) between macaques with SIB and controls. (A) Mean SC at the whole-brain level in self-injury macaques (SIB) versus controls (Con). (B) Bar plot showing significant differences in SC between lobes in self-injury macaques and those in controls. (C) Comparison of average SC between lobes for the 2 groups. (D) Bar plots showing SC for regions with significant differences between SIB and Con. (E) Visualization of brain regions with significant differences on the mid-gray surfaces of the macaque template. Red indicates regions where SC is greater in self-injury macaques, while blue indicates the opposite. (F) Mean FC at the whole-brain level in SIB versus Con. (G) Whisker plot showing significant differences in FC between lobes in SIB and those in Con. (H) Comparison of average FC between lobes for the 2 groups. (I) Bar plots showing functional connectivity density (FCD) for regions with significant differences between SIB and Con. (J) Visualization of brain regions with significant differences on the mid-gray surfaces of the macaque template. Red indicates regions where FCD is greater in self-injury macaques than in controls, while blue indicates the opposite. The abbreviations and the names of the brain regions are shown in Table S4. SIB, *n* = 3; Con, *n* = 9.

The whole-brain structural connectivity (SC) strength in the SIB group exhibited an increasing trend (*P* = 0.259) relative to that in controls (Fig. 6A). At the lobar level, we found that the self-injury macaques exhibited stronger SC between most lobes (Fig. 6C). Notably, the SCs between the parietal and occipital lobes (*P* = 0.001) and between the frontal lobe and subcortical structures (*P* = 0.005) were significantly stronger in the SIB group (Fig. 6B).

We then conducted a region-based analysis on 101 predefined brain regions, comprising 88 cortical and 13 subcortical regions in each hemisphere. Regions with a higher SC strength in the self-injury macaques were primarily located in multimodal areas such as the medial OFC, temporal pole, and parietal lobe: area 25 (*P* = 0.001), fundus intraparietal sulcus (*P* = 0.029), ERh (*P* = 0.029), granular temporal pole (*P* = 0.029), and floor of the superior temporal area (*P* = 0.029). Additionally, 12 cortical regions in the self-injury macaques exhibited a significantly higher SC strength compared with those in the control group, albeit to a lesser extent (Fig. 6D and E, uncorrected *P* < 0.05). Conversely, only 2 subcortical regions in the SIB group demonstrated reduced SC strength (uncorrected *P* < 0.05).

Using network-based statistics (NBS), we identified 79 brain region-to-region connections that were significantly stronger in the SIB than in the control macaques (shown as red lines in Fig. S2A) and 27 connections that were significantly weaker (shown as blue lines in Fig. S2A). The stronger connections in the self-injury macaques compared with those in the controls were primarily concentrated within the frontal and temporal lobes, as well as between these lobes and subcortical structures (Fig. S2B). In contrast, the weaker connections in the SIB group were mainly located between the parietal and temporal lobes (Fig. S2C).

We employed a series of GLMMs to conduct a comparative analysis of rs-fMRI between the SIB and the control macaques at 3 levels: whole brain, lobe, and specific brain regions. We calculated whole-brain mean functional connectivity (FC) as the average value of the lower triangle of the FC matrix. Our results indicated a trend toward increased mean FC in self-injury macaques, although this was not statistically significant (*P* = 0.084, Fig. 6F). Further analyses of FC differences among the 4 lobes (frontal, parietal, temporal, and occipital) and subcortical structures showed higher FC values in self-injury macaques compared with those in controls, which aligns with the observed enhancement at the whole-brain level (Fig. 6H). Specifically, self-injury macaques exhibited significantly higher FC between the parietal and frontal lobes (uncorrected *P* = 0.005), as well as between the occipital and parietal lobes (uncorrected *P* = 0.019) (Fig. 6G). At the regional level, we analyzed 101 predefined brain regions, which included 88 cortical and 13 subcortical regions per hemisphere. Consistent with prior findings, 18 regions showed higher functional connectivity density (FCD), predominantly in the parietal lobe (*n* = 8, Fig. 6I and J), including fundus intraparietal sulcus (uncorrected *P* < 0.001), area 23 (uncorrected *P* = 0.005), area 30 (uncorrected *P* = 0.006), area 7op (uncorrected *P* = 0.008), medial intraparietal area (uncorrected *P* = 0.025), anterior intraparietal area (uncorrected *P* = 0.032), V6 (uncorrected *P* = 0.040), and lateral intraparietal area (uncorrected *P* = 0.044). Additionally, significant increases in FCD were observed in 3 frontal lobe regions (area 24c prime, uncorrected *P* = 0.011; area 44, uncorrected *P* = 0.036; and area 24a/b prime, uncorrected *P* = 0.018) and one occipital lobe region (middle temporal area, uncorrected *P* = 0.040) (Fig. 6I and J).

The temporal lobe analysis revealed significant changes in FCD across 6 regions; however, only one region (caudomedial belt region, uncorrected *P* = 0.031) showed increased FCD, while 5 regions demonstrated decreased FCD (area 35, uncorrected *P* = 0.007; rostral areas of the core, uncorrected *P* = 0.016; rostral ERh, uncorrected *P* = 0.038; rostral areas of the lateral belt, uncorrected *P* = 0.039; and agranular temporal pole, uncorrected *P* = 0.044) in self-injury macaques (Fig. 6I and J). NBS identified differing FC between region pairs in self-injury macaques and those in controls. As the primary threshold increased, a stable set of differing functional connections persisted, with results at a high threshold (*t* = 4) shown in Fig. S2D. Notably, most of the increased FC in self-injury macaques was localized between the frontal and parietal lobes (*n* = 55) (Fig. S2E), while only 7 connections showed reduced strength in self-injury macaques compared with those in controls (Fig. S2F).

### Ketamine treatment had no therapeutic effect on SIB

Ketamine, as an *N*-methyl-d-aspartate receptor antagonist, has been revealed to have robust antidepressant effects and probably antisuicidal effects through impact on glutamate neurotransmission [18,19] and the FC of the subgenual anterior cingulate cortex [20]. We tested the potential treatment effect of ketamine (1.0 mg/kg, 3.5 weeks, 2 injections per week) in self-injury macaques and found no difference in wound score and behavior characteristics between prior ketamine and post-ketamine administration. Similarly, no change was found in macaques’ body weight, blood cortisol, 5-HT, and oxytocin. Therefore, a low dose of ketamine had no treatment effect on macaques with SIB.

## Discussion

Spontaneously, self-injury macaques may exhibit a potential translational value as a model for NSSI in humans, demonstrating overlapping behavioral, neurobiological, and neuroimaging features. When a battery of behavioral paradigms was conducted, the self-injury macaques exhibited similar outcomes to human NSSI, reflected by reduced locomotor activity [9] and social interaction, impaired sensory function characterized by hypoalgesia [11], emotional dysregulation [21,22], and deficient cognition and flexibility [23,24] when compared with the controls. Neurochemical analyses revealed diminished serotonergic activity, abnormal cortisol, and reduced oxytocin levels, potentially underlying emotional deficits. Structural neuroimaging showed amygdala enlargement (linked to human suicidality [25]) and reduced GMV in frontal regions (e.g., PFC and OFC), aligning with human findings of emotional dysregulation, stress hypersensitivity, and behavioral disinhibition.

Importantly, the self-injury macaques demonstrated impaired performance in the SWM and RL tasks, which have been proven to be the responsibility of the PFC [26]. A significantly high bias score, low win–stay, and high win–shift were found in self-injury macaques, suggesting that compulsivity and impulsivity might be an implicit cause of SIB, as observed in NSSI [15]. Impaired response inhibition—a core component of impulsivity—was also reflected by a shorter reaction time while making decision in self-injury macaques. Dysfunctional PFC activity was consistent with our neuroimaging results that significant reductions in the GMV including large areas of the PFC, including area 45, area 46d, and area 46v/f, were found in self-injury macaques.

HPA axis dysregulation in NSSI patients, particularly those with comorbid depression, may drive SIB via cortisol deficiency impairing cognition and impulse control [27,28]. Reduced 5-HT, critical for mood regulation, is observed in psychiatric patients and self-injury macaques [17]. Aligning with the deficit in paired social interaction, oxytocin, a neuropeptide modulating social bonding, had a tendency to have a lower level in the CSF of self-injury macaques. Oxytocin has been understudied in NSSI, although low CSF oxytocin correlates with suicidal intent [16,29]. A hypothesis posits that impaired self-perception in NSSI patients triggers social dysfunction, prompting self-injury to seek connection [24]. This study first reported lower plasma oxytocin, alongside significantly reduced cortisol and 5-HT in self-injury macaques, validating NHP models for clinical biomarker exploration in NSSI. These findings highlight intertwined neuroendocrine dysfunctions in NSSI pathology.

Metabolomic analysis may address the research gap in biomarkers for NSSI. Adolescents with NSSI exhibit reduced gut microbiota diversity [30], which correlates with the abnormal activation of digestive pathways in self-injury macaques. Dopamine is implicated in NSSI-related psychiatric mechanisms [31]. Additionally, inositol metabolite abnormalities linked to mood disorders [32] align with the observed down-regulation of phosphatidylinositol signaling in these macaques. Their distinct metabolic profiles, differing markedly from controls, mirror findings in NSSI animal models and bipolar disorder patients [17,33]. These findings suggest that metabolomic dysregulation and neural signaling disruptions may jointly underlie NSSI pathogenesis. Further confirmation should involve comparison with the human metabolome to identify the biomarkers, so as to predict disease.

In addition, we found that one male self-injury macaque showed extremely high inflammatory factors (neutrophil-to-lymphocyte ratio, monocyte-to-lymphocyte ratio, and platelet-to-lymphocyte ratio) (data not shown), which are inflammatory biomarkers linked to human suicidal behavior [34]. Another male exhibited a high testosterone/cortisol ratio (data not shown), correlating with impulsivity in male suicide attempters [35], suggesting inflammatory and neuroendocrine mechanisms in SIB. Enlarging the sample size may reduce the individual difference and highlight the biomarkers associated with SIB.

Although NSSI behaviors and biomarkers show high similarity between humans and NHPs [6], few studies have examined neuroimaging changes in self-injury macaques. Our study revealed significant brain morphometric alterations in these macaques, including subcortical structural changes, enlarged ventricles, and reduced GMV, particularly in the frontal lobe, consistent with human NSSI findings. Previous human studies associate suicidal behavior with volume changes in subcortical regions including the caudate [36], putamen [37], thalamus [38], amygdala [25], and hippocampus [39], while ventricular enlargement is linked to psychiatric disorders with high suicide risk [40]. GMV reductions in PFC, anterior cingulate cortex, and insula are also tied to self-injurious thoughts [41], which here may contribute to emotional dysregulation in macaques. Additionally, self-injury macaques exhibited enhanced SC and FC. SC increases were concentrated between parietal-occipital lobes and frontal-subcortical regions, with NBS highlighting frontal–temporal–subcortical connections—aligning with WM abnormalities in human suicidal behavior [42]. FC enhancements spanned the whole brain, notably between parietal–frontal and occipital–parietal lobes. NBS localized heightened FC to frontal–parietal networks, potentially linked to impaired executive control. Human NSSI studies describe varied fMRI patterns: moderate NSSI correlates with elevated amygdala activation to threats, while severe NSSI shows reduced amygdala–medial PFC connectivity [43]. Although animal rs-fMRI is limited by anesthesia, the widespread connectivity changes in macaques may explain multimodal neuroimaging abnormalities and guide therapeutic targets; for example, reduction of the SC and FC between the cortico-limbic network responsible for emotional response may need to be tested in macaques. The limited SC–FC overlap reflects their differing biological bases: SC represents direct anatomical pathways, whereas FC captures dynamic, multisynaptic interactions [44,45]. These neuroimaging findings align with behavioral parallels. NSSI-related impulsivity in adolescents is theorized to stem from limbic-PFC developmental imbalance [46], with structural and functional deficits in these regions contributing to self-harm [47]. Our observations of concurrent behavioral and MRI abnormalities in macaques further support their validity as models for human NSSI, offering insights into neurobiological mechanisms and potential interventions. Future investigations should extend the circuit level by activation and inhibition of certain brain regions combined with optogenetic and chemogenomic techniques and also explore physical interventions.

Our study assessed low-dose ketamine’s effect on SIB in macaques but observed no reduction in SIB severity or hormonal changes. Potential reasons may include the following: Firstly, ketamine’s rapid antidepressant/antisuicidal efficacy [18,19] may not apply here, as SIB macaques lacked overt depressive symptoms (e.g., withdrawal and low interest). Secondly, ketamine’s analgesic properties [48] might paradoxically sustain SIB in macaques with preexisting pain sensitivity dysfunction. Thirdly, intramuscular administration (vs. intravenous) could limit efficacy. Future investigations should increase macaque sample size and systematically evaluate pharmacological interventions and reveal dose-depending characteristics through comparative analysis of intravenous versus intramuscular administration routes. Notably, selecting self-injury macaques with depressive phenotypes, which hospitals often report, could enhance the efficiency and precision of translational research by establishing clinically relevant pharmacological profiles.

We cannot avoid the interspecies limitation on modeling human diseases; that is, NSSI cannot be fully manifested in animals, and basically, it is difficult to detect suicidal intent in animals for suicidal acts involves will, planning, and intention. However, just like most other human mental disorders, when we focus on every single step leading to the disorders, we can use animal models to mirror the specific aspects or traits of human diseases and assess the pathogenesis and intervention [49]. Here, the endophenotypes, e.g., neurobiological, endocrine, neuroanatomical, cognitive, and emotional measures, associated with NSSI behavior could be translatable to animal models, particularly spontaneously self-injury macaques, and may support insight into therapeutic strategies.

On the other hand, SIB, due to the pattern of repetitive stereotypies, is involved in neurodevelopmental disorders, e.g., 40% to 50% of autism spectrum disorder. In autistic youth, emotional dysregulation, higher hypersensitivity, lower intelligence quotient, and poor speech level were associated with greater severity of SIB topographies depending on different subtypes [50]. In the current experiment, we found that 2 self-injury macaques showed lower social interaction, which was consistent with autism spectrum disorder [1], also common in NSSI individuals, while approaching behavior failed to show a difference, probably because the size of the testing cage was limited.

Moreover, SIB may also be diagnosed as stereotypic movement disorder according to DSM-5 [1]. We found that self-injury macaques showed unique repetitive SIB (half leg-biting, another half arm-biting). Considering that stereotypic movement disorder is diagnosed as broader stereotypes, we recommend this macaque model to be used for studying the specific components of NSSI and developing an intervention. With artificial-intelligence-assisted precise analysis, limiting anthropomorphization by behavioral quantitative aspects may be helpful to translate behavioral research from human to animal studies.

On the other hand, SIB can be studied independently of diagnoses; for example, the Research Domain Criteria Project suggested that NSSI is likely to be clarified not by applying psychiatric diagnoses but by understanding the diverse underlying biological mechanisms that lead to this behavior. They encouraged that NSSI studies should examine neurobiological constructs (negative valence, positive valence, cognitive and social processes, and arousal/regulatory systems) across multiple units of analysis and assess how systems change across the development and course of illness [4]. It is particularly suitable for studies on animal models because animals cannot fully mimic NSSI-involved human diseases.

Other limitations include the following: The SIB biological rhythm was under a slight stress from transferring macaques into a novel environment, and the observation was over only 3 consecutive days; therefore, the stability of the biological pattern is uncertain. Future study may need to address SIB biological rhythm pattern under a naturalistic conditions including nighttime monitoring for extending the observation period and a longitudinal study across different seasons. Furthermore, we cannot avoid the intraspecies limitation due to a small sample size, leading to endophenotypical interpretation challenges. It is worthy studying the correlation between SIB severity and neuroendocrine, neuroanatomical, sensitivity, cognitive, and emotional measures and social interaction across the aspects of age and gender in large samples, providing insight into key predictors of SIB subtopography. In addition, considering that most SIB was found in the single-housed macaques, aligned with Nock and Prinstein’s dual-dimension model [51] categorizing intrapersonal (e.g., emotion regulation) and interpersonal (e.g., social) functions through negative/positive reinforcement, these macaques engage in cyclical self-injury for sensory stimulation in deprived environments. Environmental stressors or social isolation may exacerbate SIB severity. This mirrors human NSSI mechanisms where behavioral patterns serve as maladaptive coping strategies. In the future, we also need to evaluate whether these animals may reduce SIB when they are group-housed, or oppositely, the social stress may increase their SIB, to identify the environmental effects.

Finally, we propose that future research incorporate expanded macaque cohorts to cross-species integrate mechanistic data from NHP studies with human clinical data, emphasizing critical applications: (a) phenotypic refinement by dissecting subtype-specific characteristics/traits aligning with NSSI individuals and revealing the sequence of the physiological and behavioral patterns pre- and post-self-injury; (b) circuit-level analysis by utilizing advanced neuroimaging, optogenetic, and chemogenomic techniques in NHPs to map brain networks disrupted in SIB for application in precise diagnosis and prediction of the therapies; (c) physiological research and screen biomarkers by making comparison between human and animals in the central nervous and periphery systems, particular neuroendocrine and inflammatory factors, to enhance understanding of the etiology of diseases and advance targeted interventions; and (d) therapeutic research and translation by longitudinally studying various pharmacological and physical interventions on the macaque model of SIB. Pharmacological interventions involve antidepressants, atypical antipsychotics, opioid antagonists, etc. Physical interventions involve transcranial magnetic stimulation, transcranial direct/alternating current stimulation, and deep brain stimulation (DBS) As for those that have been already adopted in clinical treatments, more focused experimental research studies are required to elucidate their underlying mechanisms, especially when it comes to NHPs. Meanwhile, NHP models could be used to evaluate the effectiveness of new techniques for intervention, which cannot be conducted in humans.

Altogether, the self-injury macaque model’s ecological validity supports its utility of underlying NSSI pathogenesis of specific components, offering insights for translational research on SIB across species. Mechanistic research on NHPs remains critical to bridge gaps between preclinical and therapeutic outcomes. This highlights the need for tailored approaches when translating interventions into complex behavioral disorders like SIB, particularly in NHPs with distinct neurobiological profiles.

## Materials and Methods

For details about the materials and methods, please see the Supplementary Materials.

## Data Availability

The data are freely available upon request.

## Supplementary Materials

Supplementary 1 Materials and Methods Tables S1 to S4 Figs. S1 and S2 Movies S1 and S2 References [52–79]
