# Supplementary material for: A Study of Spontaneous Self-Injurious Behavior and Neuroimaging in Rhesus Macaques
Source: Research (Wash D C). 2025 Jul 31;8:0782. doi: 10.34133/research.0782 (PMC12311303; doi:10.34133/research.0782)
Supplement: Supplementary 1 — Materials and Methods Tables S1 to S4 Figs. S1 and S2 Movies S1 and S2 References [52–79] [file research.0782.f1.zip › Supplementary Materials_Proof_clean.pdf]

Supplementary Materials for
A study of spontaneous self-injurious behavior and neuroimaging in rhesus macaques

Ya-Li Zhang et al.

Corresponding author: Jian-Hong Wang (wangjh@mail.kiz.ac.cn), Ning Liu (liuning@ibp.ac.cn), Chun Wang (chun_wang@njmu.edu.cn)

The file includes:
Materials and Methods
Table S1 to S4
Figure S1 to S2
Legends for movie S1 to S2

Other Supplementary Material for this manuscript includes the following:
Movie S1
Movie S2

Materials and Methods

Study design

This study aimed to systematically establish behavioral, biochemical, and neuroimaging parallels between spontaneously self-injurious macaques and human non-suicidal self-injury (NSSI) populations, thereby validating this non-human primate (NHP) model for investigating underlying pathogenetic mechanisms and therapeutic interventions. Moreover, considering ketamine has rapid antidepressant effects and probably anti-suicidal properties mediated by glutamatergic modulation [19, 20], we further investigated the effect of low-dose ketamine in this animal model.

Quantitative behavioral assessments in spontaneous SIB macaques were categorized into three domains: General behavioral profiling encompassing sequential behavior patterns, biological rhythm analysis of SIB, three-dimensional (3D) movement tracking locomotor activity and paired social interaction, home-cage activity and sleep evaluation, neurological function assessment, and exploratory behavior quantification; Emotional and defensive response measured through stimulus presentation paradigms and human intruder tests (HIT); Cognitive evaluation using spatial working memory (SWM) tasks, reversal learning (RL) assessments, and win-stay-lose-shift (WSLS) strategy analysis. Biochemical profiling included plasma cortisol, serotonin (5-HT), and oxytocin levels, complemented by CSF metabolomic analysis. Multimodal neuroimaging comprised structural MRI, diffusion-weighted MRI (dMRI), and resting-state functional MRI (rs-fMRI).

Behavioral paradigms and biochemical markers were selected based on NSSI clinical presentations. Additionally, we developed a novel AI-assisted behavioral paradigm tracking system for NHP research and performed comprehensive neuroimaging analyses on self-injury macaques. This systematic integration of cross-species phenotypic data facilitates alignment between macaque biomarkers and human NSSI characteristics, ultimately establishing a translational NHP model for NSSI mechanistic exploration and therapeutic development.

Animals

We observed the sequential behaviors from ten self-injury macaques, and randomly chose three self-injury adult rhesus macaques (*Macaca mulatta*) and three age- and gender-matched control macaques (weighing 7.10±0.81 kg, 8.67±1.20 years old, two males and one female per group) from breeding colonies at the Kunming Institute of Zoology (KIZ) were used in the experiments. The macaques were housed individually under standard conditions (12 h light/dark cycle with light on from 07:00–19:00 h; humidity at 60% and temperature at 21 ± 5°C). Macaques had free access to tap water and were punctually supplied with food three times a day. Experiments were performed between 8:00 and 17:00 h. The macaque's body weight was measured every 2-4 weeks by using a transfer cage.

All experimental procedures involving animals were performed in accordance with the guidelines outlined in the Application Format for Ethical Approval for Research Involving Animals and were approved by the Institutional Animal Care and Use Committee (IACUC-PE-2023-05-001) of Kunming Institute of Zoology, Chinese

Academy of Sciences. All efforts were made to minimize animal suffering and to reduce the number of animals required.

Methods

Characterization of the self-injury macaques

Macaques exhibiting SIB were selected through experimenters' reports and confirmed by immediate repetitive SIB manifestation when observers stood and gazed near cages. These behaviors emerged spontaneously under single-housed conditions without intervention.

We conducted continuous behavioral monitoring of ten individually housed macaques over a 14-day observational period to investigate SIB patterns. For each recorded SIB episode, we performed detailed ethological analysis on 2-minute pre- and post-event intervals, systematically examining both potential trigger and consequential behavioral sequences following SIB manifestation. Further, we randomly chose three self-injury adult rhesus macaques to conduct the experiments.

The macaques underwent physical restraint via cloth limb fixation for wound assessment. Wound severity was scored according to Freeman et al. [52]: 1point =superficial <5mm; 2=superficial >5mm; 3=penetrating dermis <5mm; 4=penetrating dermis >5mm.

Behavioral experiments

Behavioral experiments were recorded by cameras and analyzed by two expert observers or using Observer™ software (Noldus Information Technology B.V., Netherlands). A single-blind procedure was used in the experiment and analyses.

Spontaneous behaviors by 3D motion-capture and behavior decomposition

A multi-view video capture device was used to record animal's spontaneous behaviors. In detail, a macaque cage (100 ×100 ×100 cm) with transparent glass walls was installed in the center of the testing room. To ensure comprehensive coverage, four cameras (Intel RealSense D435) were strategically mounted on supporting pillars, 1.1 meter from the cage, with angles meticulously adjusted to capture a clear and unobstructed view of the entire experimental arena from each camera's perspective. The macaque was gently moved into the behavior testing cage by using a transfer box and its behavior images were simultaneously acquired by four cameras at a frame rate of 30 Hz, with a resolution of 848 × 480 pixels.

The video data collection and analyses were managed by the BehaviorAtlas NHP Explorer and Analyzer software (version 1.01, Shenzhen Bayone BioTech Co.) [53]. A total of 21 key body parts of the macaque were labeled from 4200 frames from eight normal macaques to train a reliable model for identifying experimental macaque's body parts, in which 46 kinematic parameters were extracted and 14 behavioral sequences were constructed based on the parameters. The macaques' behaviors with/without apparent displacement (AD/NAD) were categorized and analyzed individually. Principal component analysis (PCA) was performed on the standardized kinematic parameters, with the cumulative proportion of variance explained by the first principal component (PC1) and the second principal component

(PC2) accounting for 96.00%. The results were standardized using Z-scores. We recorded the spontaneous behaviors of each macaque for 35 min per session, with a total of two sessions with an inter-trial interval (ITI) of a couple of days.

In addition, the self-injury macaque was paired with a gender- and age-matched stranger partner macaque and recorded the social interaction for 35 min per session.

In order to minimize inter-individual difference, each macaque was paired with two strangers × two sessions totally. Each macaque was identified and tracked by using an Anti-drift Pose Tracker (ADPT) [54].

The rump presenting [55], gaze following, approaching behavior, characterized as socially affiliative behaviors, was analyzed according to the following conditions:

Rump presenting behavior: a posture involving a stance on all limbs with the hind quarters elevated and the tail raised, directed at the partner. Gaze following behavior: one macaque looks at the other. Approaching behavior: two macaques sit or stand together, or one macaque approaches to the other with a distance between less than 30 cm within at least 15 consecutive frames (1 second).

In this experiment, two self-injury macaques and three controls were tested, because one male self-injury macaques showing elevated neutrophil-to-lymphocyte ratio (NLR), monocyte-to-lymphocyte ratio (MLR), and platelet-to-lymphocyte ratio (PLR) died.

Home-cage activity and sleep

The ActiGraph GT9X Link (3.5×3.5×1 cm, 14 grams, ActiGraph Corp, USA) was installed on the macaque's collar and used to evaluate the macaque's diurnal activity (timeframe of 7:00–19:00 and nocturnal sleep 19:00-7:00) at the home cage for consecutive eight days (one-day accommodation and middle six-day data analysis). The data were analyzed with the accompanying software ActiLife 6 (ActiGraph Corp., LLC) using a defined time period of 60 sec (epochs). The following activity measures were calculated by two time frames: daily average and maximum VMC (Vector magnitude counts), total activity counts of bouts and sleep (total sleep time, efficiency and sleep fragmentation index).

SIB biological rhythm pattern

Macaques were transferred to a novel observation chamber (100×100×100 cm) for behavioral recording by Kinect 2.0 cameras, during 10:00 - 12:30 a.m. and 14:00 - 16:30 p.m., respectively, over three days. Every middle 30-minute video was analyzed.

Neurological function

Neurological function of macaques was evaluated using a developed neurologic deficit score from our previous study [56], which assigned scores to the motor system (16 points), skeletal muscle coordination (9 points), and the sensory system (25 points), that were tabulated to a total of 50 possible points. Score 0 corresponded to normal behaviors, while higher scores represented neurological deficits. The control animals were 18 adult health macaques.

Exploratory behavior test

We designed an exploratory test in which a neutral fishbone-shaped laser icon (length 11.5 cm, height 4.5 cm) was presented into the macaque's cage by a familiar experimenter positioned 1m front-center, avoiding eye contact. The laser was

executed three 1-min phases: clockwise/counterclockwise cage perimeter movements (5 sec/round) and vertical shaking 5-10 cm near the macaque's abdomen, maintained non-contact to the animal. A Sony HDR-CX405 camera recorded behaviors, with two expert observers scoring via Observer™ software using standardized ethograms. The duration and frequency of macaques' mouth licking, hand touching, and body following with eyes watching were recorded.

Emotional response test

Food preference assessment in macaques was conducted using a Wisconsin General Test Apparatus (WGTA) with a transparent box (55 cm× 20 cm×15 cm). Four food types (hawthorn candy, peanut, raisin, sweet potato) were semi-randomly paired (36 trials/session ×3 sessions), with selections recorded. Subsequently, an emotional response test was conducted modifying from Pujara et al [57]. Novelty response trials (12 trials/session ×3 sessions at two-day intervals) introduced four stimuli (striped rope, plastic tube holder, rubber snake/spider) in the transparent box with favorite food rewards on the top center of the box. Stimuli appeared semi-randomly (2 presentations each) alongside null trials (4/session), maintaining 30-second ITI. The maximum of stimulus presentation was 60 s, with snake toys excluded from initial quadrants and consecutive presentations.

Human intruder test (HIT)

Defensive response to human intruder was tested according to Kalin and Shelton [58], across three sessions over six months. Macaques in relocated cages underwent three sequential 5-minute exposures to unfamiliar intruders: profile orientation with no eye contact (NEC), direct eye contact (EC), and back presentation, each preceded by 3-min acclimation. Intruders maintained 1.0m distance from the cage center during static postures. Affective reactivity to human intruder was scored based on locomotion, stereotypy, defensive position in cage (head in cage's rear half), withdrawal behaviors (freezing, lipsmacking, grimacing, and self-directed biting behaviors), vigilance, aggression (threat, cage shaking behaviors). Particularly, vigilance referred to the duration of the macaque showing attention to and looking at the intruder except of aggressive behavior, while threat referred to the frequency of at least two of the following behaviors directed towards the intruder: intense staring with eyes wide open, lips parted in o shape, head-bob, ear pulled back, bark vocalization or lunges [59, 60].

Spatial working memory (SWM) and Reversal learning (RL)

The SWM was performed by using the WGTA [61]. Six delay lengths (A–F) were semi-randomly assigned per session: A = B×0 s, B = B×1 s, C = B×2 s, D = B×3 s, E = B×4 s, F = B×5 s. Macaques underwent 3-day habituation, followed by B=0 s training until achieving ≥91.67% accuracy (33/36 trials) for 5 consecutive sessions or max 20 sessions. Subsequent B=3 s training lasted 20 sessions. The percentage of correct choice at B=0s/3s and sessions-to-criterion were recorded.

For discrimination learning, macaques distinguished rewarded circles (S+) from non-rewarded diamonds (S−) in 36-trial sessions. After reaching ≥88.89% accuracy (32/36 trials) for 3 sessions (max 20), RL commenced by switching rewards to diamonds. Training continued until the same accuracy criterion or 20-session limit. The percentage of correct choice at each session and sessions-to-criterion were

analyzed. Side preference during RL was calculated as: [(preferred choices−nonpreferred choices)/total choices]×100, reflecting stereotypical behavior.

Win-stay-lose-shift (WSLS)

On the preceding trial of WSLS, reinforcement was provided (win), and the same stimulus should be chosen on the next trial (stay). However, if reinforcement did not occur (lose), on the next trial the other stimulus should be chosen (shift). Usually WSLS was tested in midsession reversal tasks in animals, reflecting the cognitive flexibility, decision-making and impulsivity of animals [62]. Here, WSLS was analyzed with MATLAB programming for code generation, including the percentage of the win-stay/shift and lose-stay/shift for the macaques performing the acquisition and RL process.

Measurement of cortisol, 5-HT and oxytocin in plasma

The macaques were fasted breakfast in the morning and 1.5 mL blood was quickly collected from the macaque's femoral vein without sedation within a few minutes to minimize effects of the sampling procedure itself on the cortisol level. The cortisol, oxytocin and 5-HT were measured by using QuicKey Pro macaque Cortisol ELISA Kit (Elabscience Biotechnology, E-OSEL-MK0002), Oxytocin ELISA Kit (Elabscience Biotechnology, E-EL-0029) and Serotonin ELISA Kit (Elabscience Biotechnology,E-EL-0033), respectively. Each sample was measured in triplicate repetitions.

Targeted metabolomics

We collected cerebrospinal fluid (CSF) samples from the interspace between the last two lumbar with a sterilized 22-gauge puncture needle in macaques under anesthesia with ketamine (10 mg/kg). The CSF was centrifuged at 4℃, 1489 g for 15 min. The supernatant was immediately collected and stored at −80°C until analysis. Metabolomic analysis of CSF was based on LC/MS. Briefly, the analysis of hydrophilic metabolites and lipids (H650 and HL2400) were performed on a UHPLC system (LC-30AD, Shimadzu) coupled with QTRAP MS (6500+, Sciex) at Shanghai Applied Protein Technology Co., Ltd.. The analytes were separated using a HILIC column (Phenomenex, Luna NH2, 2.0 mm × 100 mm, 3 μm) and a C18 column (Phenomenex, Kinetex C18, 2.1 × 100 mm, 2.6 μm). The measured metabolites were quantified using Multi Quant or Analyst software.

Neuroimaging acquisition and analysis

Macaques were premedicated with atropine (0.05 mg/kg, i.m.) and ketamine (10 mg/kg, i.m.). Anesthesia was maintained with continuous intravenous propofol at 15 mg/kg/h. We monitored end-tidal carbon dioxide (ETCO2) levels and respiratory rates using an MRI-compatible system and covered the animals with a blanket to prevent hypothermia. To ensure a reliable comparison between the SIB and control group, we increased the controls to nine age- and gender-matched healthy macaques.

Using a 3.0 T UMR790 MRI scanner at the KIZ, we obtained MRI, dMRI, and rs-fMRI data. The macaques were under general anesthesia to reduce stress and

motion artifacts, consistent with previous findings that resting-state functional activity persists under anesthesia in both humans and macaques [63, 64].

Structural MRI Data Acquisition

T1-weighted images were acquired using a 3D fast spoiled gradient echo sequence (voxel size = 0.5 mm isotropic, TE = 5.6 ms, TR = 13.01 ms, flip angle = 8°). T2-weighted images were collected with a fast spin echo sequence (voxel size = 0.5 mm isotropic, TE = 396.48 ms, TR = 3400 ms, flip angle = 59°), using a 12-channel head coil.

dMRI Data Acquisition

dMRI parameters included TR = 7740 ms, TE = 90 ms, flip angle = 90°, field of view = 96 mm, matrix size = 96 × 96, and slice thickness = 1 mm, resulting in a voxel resolution of 1 × 1 × 1 mm^3. Diffusion weighting was applied with b-values of 1000 and 2000 s/mm^2 across 64 directions, plus one non-diffusion-weighted image.

Rs-fMRI Data Acquisition

Rs-fMRI images were obtained using an echo-planar imaging sequence with a voxel size of 1.5 mm isotropic, TE of 29 ms, TR of 1700 ms, and a flip angle of 80°. Each session included 500 EPI volumes, with reverse phase encoding data collected to aid in image correction.

Structural MRI Data Analysis

We processed structural data using AFNI [65], FSL [66], ANTs [67], and FreeSurfer [68]. Each animal's T1 image was registered to the NIMH Macaque Template (NMT, version 2.0) [69]. An in-house neural network generated initial skull stripping and WM masks. T2 images were co-registered with T1 using rigid-body transformation, followed by bias correction. A FreeSurfer-based pipeline produced WM and GM surfaces, which were manually reviewed and adjusted for accuracy.

Brain surfaces were segmented into four lobes and 88 regions per hemisphere using CHARM1 and CHARM5 atlases [69]. FreeSurfer extracted GMV for each region. Subcortical regions were parcellated using the SARM atlas [70], covering 13 nuclei like the amygdala and thalamus. Segmentation was done by applying the registration matrix inversely to the SARM atlas. Ventricles were segmented similarly, with manual checks as needed. Volumes of all regions, including ventricles, WM, and subcortical structures, were calculated based on voxel counts.

dMRI Data Analysis

We processed dMRI images using the HCP-NHP diffusion pipeline. Preprocessing included B0 intensity normalization, EPI distortion correction, eddy current and motion correction, and alignment to T1 images. Using MRtrix, we estimated tissue-specific response functions and calculated fiber orientation distributions with the constrained spherical deconvolution model [71]. Fractional anisotropy, axial diffusivity, radial diffusivity, and apparent diffusion coefficient were computed [72]. Probabilistic fiber tractography was performed with MRtrix, using anatomically constrained tractography, with default parameters. Biases were reduced using the SIFT2 algorithm [73]. The brain was parcellated with CHARM5 and SARM atlases. We constructed a weighted 202 × 202 structural connectivity (SC) matrix with MRtrix's tck2connectome, normalizing edge weights using the -scale_invnodevol

option [74]. Connections were validated only if present in all 12 macaques. SC was analyzed at three levels: for the whole brain, it was calculated as the sum of all connections; for each lobe, it was defined as the sum of connection strengths between regions within that lobe; and for individual brain regions, it was measured as the sum of their connection strengths with all other brain regions.

Rs-fMRI Data Analysis

We preprocessed rs-fMRI data using AFNI, following established workflows [75]. The data were divided into five segments of 100 TRs each. The brain was parcellated using CHARM5 and SARM atlases, consistent with structural analysis procedures. Functional connectivity (FC) networks were computed by calculating Pearson's correlation coefficients between mean time series of brain region pairs, forming a 202 × 202 matrix. These matrices were then converted to z-scores using Fisher's z-transformation.

Whole-brain FC was assessed by averaging the lower triangle of the FC matrix, including both positive and negative correlations. For lobe-level analysis, the 202 regions were grouped into lobes and subcortical nuclei for each hemisphere, aggregated into two 5 × 5 matrices. Functional connectivity density (FCD) for each region was calculated by averaging its connections with all other regions, setting negative connections to zero [76].

Network-Based Statistic (NBS) Approach

We used NBS approach to identify clusters of regions with differential FC and SC within intra-hemisphere networks [77]. This non-parametric method controls family-wise errors in multiple comparisons. Connected graph components exceeding thresholds (FC: $t = 3.0$ to 4.0; SC: $t = 2.9$ to 3.5) were tested for significance against a null distribution using permutation testing. The NBS framework enhances statistical power by rejecting the null hypothesis at the component level instead of the individual edge level. Analyses were performed with the NBS toolbox and MATLAB R2021b, and results were visualized with BrainNet Viewer [78].

Administration with ketamine

A low dose of hydrochloric acidulated ketamine (1.0 mg/kg, Jiangsu Zhongmubeikang Pharmaceutical Company) was intramuscularly (i.m.) injected to the two SIB and three control macaques respectively in the morning (divided into four injections with an interval of 10 min, on every Monday and Thursday) [79], total seven × four injections. Blood collection and behavioral tests were conducted prior to and post ketamine administration. The self-injury and control macaques were moved into a novel room with a 24 hr-recording camera in front of each cage, for five days, prior to and post ketamine administration. The wound and neurological function were measured prior to the ketamine administration and post the behavioral test. We also collected plasma in macaques prior to and post the ketamine administration to quantify the levels of cortisol, oxytocin and 5-HT.

Statistical analysis

Behavioral and physiological data was expressed as mean ± standard error of the mean (S.E.M) and analyzed using the GraphPad Prism v8.00 (GraphPad Software, La

Jolla, CA, United States). For physiological and behavioral experiments, Shapiro-Wilk test was used for normality test. Unpaired *t*-test (two-tailed), Analysis of variance (ANOVA) test (with repeated measures where appropriate), or Mann–Whitney U test was applied respectively depending on whether the data were normally distributed or not. Differences were considered significant when $p \leq 0.05$.

For metabolomics data, univariate and multivariate statistical analysis were employed to examine the processed data between the self-injury and control macaques, including principal component analysis (PCA), orthogonal partial least squares discriminant analysis (OPLS-DA), followed by KEGG pathway analysis. Statistical difference was determined using an unpaired *t*-test (two-sample, uncorrected), and $p < 0.05$ indicated statistical significance.

For MRI data analyses to assess the structural differences between SIB and control macaques, we employed Generalized Linear Mixed Models (GLMMs) to analyze measurements at the global, lobe, and region levels, incorporating Hemisphere as a random factor. Importantly, all structural data were adjusted for the intracranial volume of the corresponding hemisphere. For the evaluation of SC differences, Hemisphere was included as a random factor in the GLMM. For the evaluation of FC differences, we analyzed whole-brain FC differences using the GLMM, treating Segment as a random factor. At the lobar and regional levels, we conducted GLMMs with both Segment and Hemisphere included as random factors. Given the relatively small sample size of our study, we have presented both corrected and uncorrected significant results to provide a comprehensive overview of our findings. We note here that all p values are corrected for the number of lobes or regions using the false discovery rate (FDR) method unless specified otherwise.

Supplementary Tables

Table S1. Differential hydrophilic metabolites in CSF

Name	Class	Mass	RT	fold change	p-value
Arachidic Acid	Fatty Acyls	311.3/311.3	12.10	1.0534	0.0351
Chenodeoxycholic acid (CDCA)	Steroids and steroid derivatives	391.4/391.401	9.28	6.1968	0.0450
Deoxycholic acid (DCA)	Steroids and steroid derivatives	391.4/391.402	9.31	2.5292	0.0344
Gluconic acid	Organooxygen compounds	195/129	7.80	0.6600	0.0153
Malic acid	Hydroxy acids and derivatives	133/115.1	8.55	0.8635	0.0350
Maltotriose	Organooxygen compounds	522.2/325.0	8.84	2.3388	0.0456
Myo-inositol	Organooxygen compounds	179/161	7.72	0.6319	0.0051
N-Acetylneuraminic acid	Organooxygen compounds	310.1/121.0	7.75	0.6494	0.0005
Tyrosine	Carboxylic acids and derivatives	182.1/136.1	4.98	0.6732	0.0377

fold change > 1 indicates increased metabolites and < 1 indicates decreased metabolites.

Table S2. Differential lipids in CSF

Name	Class	CalMz	RT	fold change	p-value
PA(18:1/20:1)	Phosphatidic acid	727.5/309.3	5.8	0.8355	0.0315
PC(14:0/20:4)	Phosphatidylcholine	812.5/303.2	5.86	1.3223	0.0085
PC(15:0/18:3)	Phosphatidylcholine	800.5/241.2	5.86	1.2181	0.0110
PC(15:0/22:5)	Phosphatidylcholine	852.6/241.2	5.86	1.1995	0.0137
PS(18:1/18:1)	Phosphatidylserine	786.5/281.2	11.42	1.5596	0.0286
DG(18:2/22:4)	Diacylglycerol	686.6/337.3	5.6	0.6490	0.0496
DG(18:3/20:1)	Diacylglycerol	662.6/335.3	5.65	0.6778	0.0199
DG(19:0/19:0)	Diacylglycerol	670.6/355.3	7	1.3296	0.0334
TG(45:1)-FA16:0	Triglycerides	780.7/507.4	8.41	0.8859	0.0205
TG(45:1)-FA18:1	Triglycerides	780.7/481.4	8.41	0.8817	0.0198
TG(46:3)-FA18:3	Triglycerides	790.7/495.4	7.91	0.7079	0.0437
TG(47:1)-FA18:1	Triglycerides	808.7/509.4	8.71	0.8404	0.0377
TG(48:2)-FA16:0	Triglycerides	820.7/547.4	8.69	0.8246	0.0288
Cer(d18:1/22:1)	Ceramide	620.7/264.4	5.7	0.5407	0.0364

TG(49:3)-FA18:2	Triglycerides	832.8/535.5	8.58	0.8405	0.0039
TG(50:6)-FA20:4	Triglycerides	840.7/519.4	7.73	0.7459	0.0399
TG(51:1)-FA17:0	Triglycerides	864.8/577.5	9.59	0.8826	0.0410
TG(51:5)-FA18:3	Triglycerides	856.8/561.5	8.31	0.7106	0.0280
TG(52:4)-FA20:3	Triglycerides	872.8/549.5	8.87	0.8372	0.0078
TG(53:1)-FA16:0	Triglycerides	892.8/619.5	10.03	0.7107	0.0139
TG(54:5)-FA22:4	Triglycerides	898.8/549.5	8.96	1.3055	0.0366

fold change > 1 indicates increased metabolites and < 1 indicates decreased metabolites.

Table S3. Analysis of KEGG pathway in self-injury macaques

**Pathway**	**p-value**	**Upregulation or downregulation**
**The analysis of hydrophilic metabolites**		
carbohydrate digestion and absorption	0.0405	upregulation
bile secretion	0.0089	upregulation
secondary bile acid biosynthesis	0.0013	upregulation
dopaminergic synapse activity	0.0182	downregulation
phosphatidylinositol signaling system	0.0434	downregulation
melanogenesis	0.0091	downregulation
prolactin signaling	0.0167	downregulation
taste transduction	0.0478	downregulation
glucagon signaling	0.0390	downregulation
pyruvate metabolism	0.0463	downregulation
citrate cycle	0.0301	downregulation
methane metabolism	0.0071	downregulation
carbon metabolism	0.0122	downregulation
**The analysis of lipids**		
alpha-linolenic acid metabolism	0.0439	upregulation
linoleic acid metabolism	0.0282	upregulation
metabolism of glycine,serine, and threonine	0.0498	upregulation
phosphatidylinositol signaling system	0.0291	downregulation
sphingolipid metabolism	0.0252	downregulation
necroptosis	0.0101	downregulation
**The following pathway changes were not significant**		
glycerophospholipid metabolism	ns	upregulation
retrograde endocannabinoid signaling	ns	upregulation
neurotrophin signaling	ns	downregulation

cAMP signaling	ns	downregulation
sphingolipid signaling	ns	downregulation
phospholipase D signaling	ns	downregulation
glycerolipid metabolism	ns	downregulation
FcγR-mediated phagocytosis	ns	downregulation
adipocytokine signaling	ns	downregulation
GnRH signaling	ns	downregulation
fat digestion and absorption	ns	downregulation

Table S4. The abbreviation and region name of the brain areas in the figures of neuroimaging results

Abbreviation	Region Name
Acb	accumbens
AI	primary auditory cortex
AIP	anterior intraparietal area
AL/RTL	rostral areas of the lateral belt
Amy	amygdala
ant STSf	anterior fundus of the superior temporal sulcus
ant TE	anterior area TE
area 10	frontal pole
area 11	area 11
area 12m/o	medial and orbital area 12
area 12r/l	rostral and lateral area 12
area 13	area 13
area 14	gyrus rectus
area 23	area 23
area 24a/b	areas 24a and 24b
area 24a/b prime	areas 24a' and 24b'
area 24c	area 24c
area 24c prime	area 24c'
area 25	subgenual cortex
area 29	area 29
area 30	area 30
area 31	area 31
area 32	area 32
area 35	area 35
area 36	area 36
area 3a/b	areas 3a and 3b
area 44	area 44
area 45	area 45

area 46d	dorsal area 46
area 46v/f	ventral and fundus portion of area 46
area 5d	dorsal area 5
area 7a/b	inferior parietal lobule areas 7a and 7b
area 7m	area 7 (PGm) on the medial wall
area 7op	parietal operculum
area 8A	periarcuate area 8A (frontal eye fields)
area 8B	area 8B
area 9	area 9
area v23	area v23
areas 1-2	areas 1-2
caudal ERh	caudal entorhinal cortex
Cd	caudate
CL/ML	caudal areas of the lateral belt
CM	caudomedial belt region
EpiThal	epithalamus
Fro	frontal lobe
Fro-Sub	frontal-subcortical
FST	the floor of the superior temporal area
fundus IPS	fundus of the intraparietal sulcus
G	gustatory cortex
HF	hippocampal formation
Hy	hypothalamus
Iam/Iapm	medial agranular insular region
Ins	insula
lat Ia	lateral agranular insular region
LIP	lateral intraparietal area
LOP	lateral occipital parietal area
LVPal	lateral and ventral pallium
M1	primary motor cortex
Mid	midbrain
mid ERh	middle entorhinal cortex
MIP	medial intraparietal area
MST	medial superior temporal area
MT	middle temporal area
Occ	occipital lobe
OLF	primary olfactory cortex
Par	parietal lobe
parabelt	parabelt areas of auditory cortex
Par-Occ	parietal-occipital
Pd	pallidum

PEa	area PEa
Pi	parainsula
PMd	dorsal premotor cortex
PMv	ventral premotor cortex
post TE	posterior area TE
PrCO	precentral operular area
preSMA	presupplementary motor area
PreThal	prethalamus
PrT	Pretectum
Pu	putamen
R/RT	rostral areas of the core
Ri	retroinsula
RM/RTM	rostral areas of the medial belt
rostral ERh	rostral entorhinal cortex
RTp	polar rostrotemporal cortex
SII	secondary somatosensory cortex
SMA	supplementary motor area
STGr	rostral superior temporal gyrus
Sub	subcortical
TAa	area TAa
TE in STSv	area TE in the ventral STS
Tem	temporal lobe
TEO	area TEO
TF/TFO	areas TF and TFO
TGa	agranular temporal pole
TGd	dysgranular temporal pole
TGg	granular temporal pole
TH	area TH
Thal	thalamus
TPO	temporal parietooccipital associated area
Tpt	temporo-parietal area
V1	primary visual cortex
V2	visual area 2
V3d/V3A	visual areas V3d and V3A
V3v	ventral visual area 3
V4d	dorsal visual area 4
V6	visual area V6
V6A	visual area V6A

Supplementary Figure 1

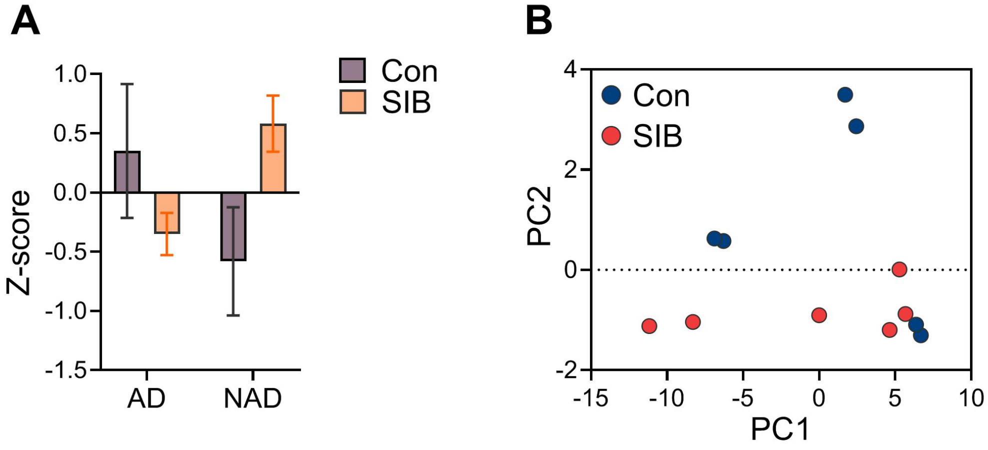

Figure S1. Spontaneous behavior in macaques.

A: Self-injury macaques showed overall low apparent displacement (AD) and high non-apparent displacement (NAD) when compared with the controls.

B: Principal component analysis (PCA) of kinematic parameters between self-injury macaques and control animals.

Supplementary Figure 2

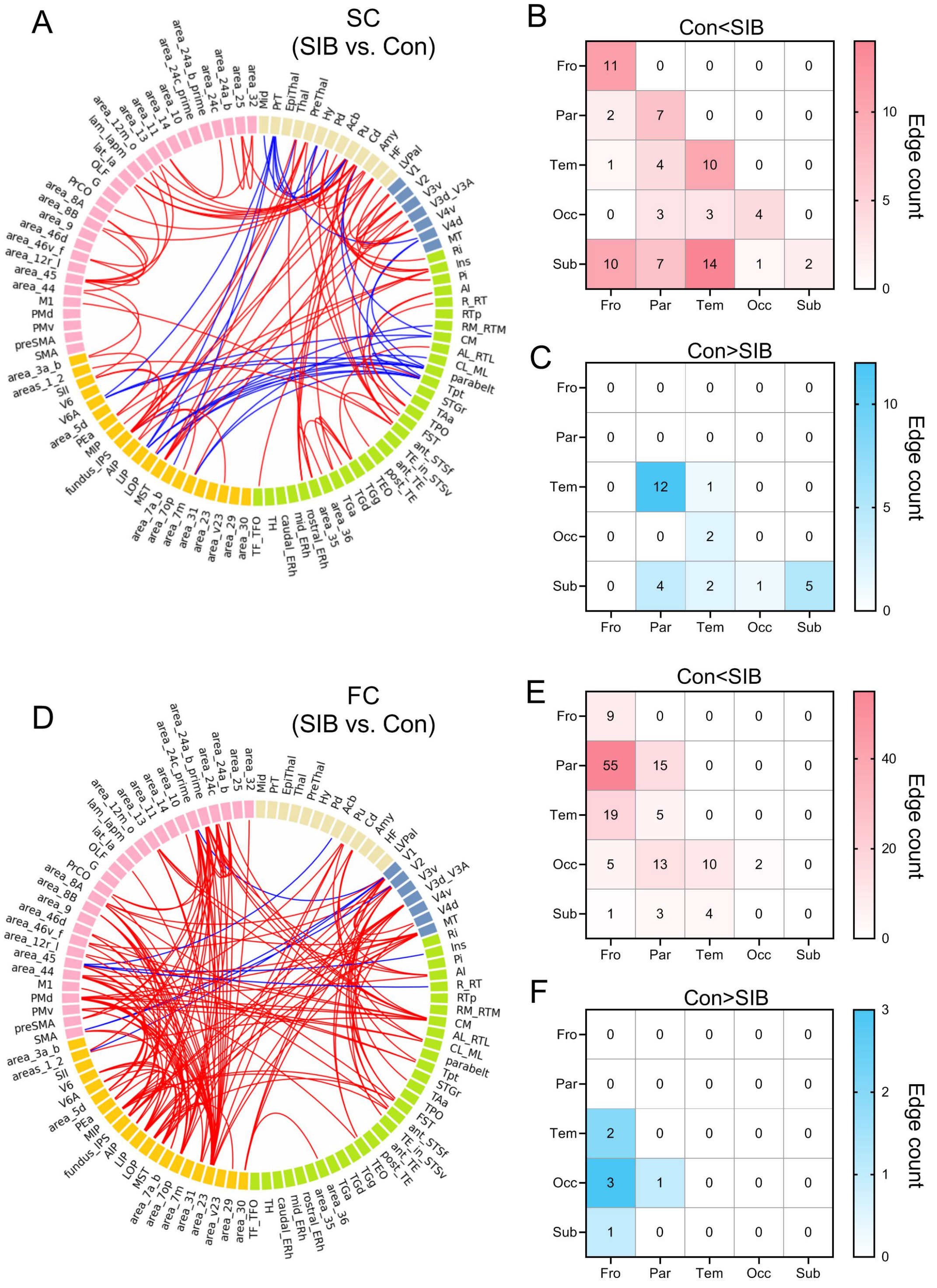

Figure S2. NBS results of structural and functional connectivity

A & D: Visualization of structural (A) and functional (D) connectivity changes, with decreases shown in blue and increases in red, in self-injury macaques (SIB) compared with controls (threshold of FC: t = 4.0; threshold of SC: t = 2.9). B-C: Heatmaps showing the number of increased (B) and decreased (C) structural connectivity

instances in self-injury macaques compared with controls at the threshold of t = 2.9. Fro - frontal lobe; Par - parietal lobe; Tem - temporal lobe; Occ - occipital lobe; Sub - subcortical area.
E-F: Heatmaps depicting the number of increased (E) and decreased (F) functional connectivity instances in self-injury macaques compared with controls at the threshold of t = 4.

Legends for movie S1 to S2

Movie S1 showed a self-injury macaque quietly biting its arm.
Movie S2 showed a self-injury macaque biting its lower limbs engaging in stereotypical behavior.
